# ^1^H, ^13^C, and ^15^N backbone chemical-shift assignments of SARS-CoV-2 non-structural protein 1 (leader protein)

**DOI:** 10.1007/s12104-021-10019-6

**Published:** 2021-03-26

**Authors:** Ying Wang, John Kirkpatrick, Susanne zur Lage, Sophie M. Korn, Konstantin Neißner, Harald Schwalbe, Andreas Schlundt, Teresa Carlomagno

**Affiliations:** 1 Centre of Biomolecular Drug Research (BMWZ), Leibniz University Hannover, Schneiderberg 38, 30167 Hannover, Germany; 2 Group of NMR-Based Structural Chemistry, Helmholtz Centre for Infection Research, Inhoffenstrasse 7, 38124 Braunschweig, Germany; 3 Institute for Molecular Biosciences, St Lucia, QLD 4072 Australia; 4 Institute for Organic Chemistry and Chemical Biology, 60438 Frankfurt, Germany; 5 Center for Biomolecular Magnetic Resonance (BMRZ), Johann Wolfgang Goethe-University Frankfurt, Max-von-Laue-Str. 7, 60438 Frankfurt, Germany

**Keywords:** SARS-CoV-2, Nsp1, Non-structural proteins, New drug targets, 5′ untranslated region, NMR spectroscopy

## Abstract

The current COVID-19 pandemic caused by the Severe Acute Respiratory Syndrome Coronavirus 2 (SARS-CoV-2) has become a worldwide health crisis, necessitating coordinated scientific research and urgent identification of new drug targets for treatment of COVID-19 lung disease. The *covid19-nmr* consortium seeks to support drug development by providing publicly accessible NMR data on the viral RNA elements and proteins. The SARS-CoV-2 genome comprises a single RNA of about 30 kb in length, in which 14 open reading frames (ORFs) have been annotated, and encodes approximately 30 proteins. The first two-thirds of the SARS-CoV-2 genome is made up of two large overlapping open-reading-frames (ORF1a and ORF1b) encoding a replicase polyprotein, which is subsequently cleaved to yield 16 so-called non-structural proteins. The non-structural protein 1 (Nsp1), which is considered to be a major virulence factor, suppresses host immune functions by associating with host ribosomal complexes at the very end of its C-terminus. Furthermore, Nsp1 facilitates initiation of viral RNA translation via an interaction of its N-terminal domain with the 5′ untranslated region (UTR) of the viral RNA. Here, we report the near-complete backbone chemical-shift assignments of full-length SARS-CoV-2 Nsp1 (19.8 kDa), which reveal the domain organization, secondary structure and backbone dynamics of Nsp1, and which will be of value to further NMR-based investigations of both the biochemical and physiological functions of Nsp1.

## Biological context

The ongoing COVID-19 pandemic has initiated intense scientific research into the causative pathogen, severe acute respiratory syndrome coronavirus 2 (SARS-CoV-2). Previous studies have demonstrated that SARS-CoV-2 shows close sequence homology to SARS-CoV-1, which was responsible for the SARS epidemic in 2003, and also to MERS-CoV, which causes Middle-Eastern Respiratory Syndrome (Benedetti et al. 2020; A. Wu et al. 2020a, b). With a high transmissibility and potential to induce life-threatening acute respiratory distress syndrome, SARS-CoV-2 represents a severe threat to human health worldwide. Rapid identification and characterization of druggable protein targets within the SARS-CoV-2 genome is the first step towards development of targeted therapeutic treatments for COVID-19, which together with vaccination approaches, will be critically important in mitigating the impact of the pandemic.

SARS-CoV-2 belongs to the family of beta-coronaviruses, with an enveloped, positive-sense single-stranded RNA genome (Gorbalenya et al. 2020), which encodes two large overlapping open reading frames (ORF1a and ORF1b) at the 5′-end, as well as four structural proteins and eight accessory proteins at the 3′-end (Lim et al. 2016; Zhou et al. 2020). After cell entry, the virus exploits the host translational machinery to produce the polypeptides corresponding to ORF1a and ORF1b. The polypeptides are then proteolytically cleaved into 16 functional non-structural proteins (Khailany et al. 2020), most of which are involved in assembling the host–viral replication/transcription complex (Masters 2006). Among these proteins, non-structural protein 1 (Nsp1), also known as the leader protein, plays a key role in hampering host gene expression.

SARS-CoV-2 Nsp1, the first N-terminal cleavage protein from the replicase polyprotein, is 180 residues in length, and shares ~ 85% amino-acid sequence identity with the homologous protein from SARS-CoV-1. SARS-CoV-1 Nsp1 has been structurally characterized using solution-state NMR methods, leading to an atomic-resolution structure of the globular domain comprising residues 13–128 and revealing short N-terminal and long C-terminal tails (residues 1–12 and 129–179, respectively) that are flexibly disordered (Almeida et al. 2007). Recent cryo-electron microscopy (cryo-EM) studies on Nsp1 from SARS-CoV-2 have demonstrated that host protein translation inhibition by Nsp1 is mediated by insertion of its C-terminus into the entrance of the mRNA tunnel in the small ribosomal subunit (Schubert et al. 2020; Thoms et al. 2020). In addition, it was shown biochemically that the 5′ UTR of SARS-CoV-2 is capable of promoting translation initiation of viral mRNAs through binding to the N-terminal domain (NTD) of Nsp1 (Schubert et al. 2020). However, the precise mechanism by which SARS-CoV-2 escapes from the Nsp1-mediated translation inhibition—thereby switching the host translation machinery from host to viral protein synthesis—and which specific region of SARS-CoV-2 5′ UTR binds to the NTD of Nsp1 are still unclear. Due to its dual role in inhibition of host-protein translation and stimulation of viral-protein translation, Nsp1 has been proposed as an attractive drug target for the treatment of COVID-19.

The research consortium *covid19-nmr* seeks to rapidly and publicly support the search for anti-viral COVID-19 drugs using an NMR-based screening approach that in the initial stage involves the production of all druggable proteins and RNAs from SARS-CoV-2 and their subsequent NMR chemical-shift assignment, followed by a second stage of solution-structure determination, drug-library screening and rational structure-based drug-design. Here we report the near-complete backbone assignment of full-length SARS-CoV-2 Nsp1 that represents the first step towards its structural characterization and provides a basis for residue-resolved drug-screening and protein–RNA interaction studies.

## Methods and experiments

### Construct design

The Nsp1 protein sequence studied here derives from the SARS-CoV-2 genomic sequence corresponding to NCBI GenBank entry NC_045512.2, which is identical to GenBank entry MN908947.3 (F. Wu et al. 2020a, b). A codon-optimized expression construct of SARS-CoV-2 full-length Nsp1 was inserted into the vector pETM-11 (EMBL bacterial expression vector database), containing an N-terminal His_6_-tag, and a tobacco etch virus (TEV) cleavage site. Due to the nature of the TEV cleavage site, two artificial N-terminal residues (Gly[–1] and Ala[0]) are included in the cleaved protein, before the native Nsp1 sequence starts at Met[1] (corresponding to NCBI GenPept entry YP_009725297.1).

For the NTD of SARS-CoV-2 Nsp1, domain boundaries were defined in analogy to the available NMR structure (PDB codes 2hsx/2gdt (Almeida et al. 2007)⁠) of the SARS-CoV-1 homologue Nsp1 (85% sequence identity). The expression construct contained residues 13–127 of the Nsp1 sequence. The construct was amplified from the full-length Nsp1 gene, obtained as an *E. coli-*codon-optimized DNA construct from GenScript Biotech (Netherlands). It was inserted into the pET3b-based vector pKM263, containing an N-terminal His_6_-tag, a GST-tag and a tobacco etch virus (TEV) cleavage site. Four artificial N-terminal residues (Gly[–3], Ala[–2], Met[–1] and Gly[0]) remained after proteolytic TEV cleavage, resulting in a 12.9 kDa protein.

### Protein expression and purification

Large-scale expressions of uniformly ^13^C,^15^ N-labelled full-length Nsp1 in *E*. *coli* BL21 (DE3) cells were carried out in M9 minimal medium, containing 1 g/L ^15^NH_4_Cl (Cambridge Isotope Laboratories), 2.5 g/L ^13^C_6_-D-glucose (Cambridge Isotope Laboratories) and 50 μg/mL kanamycin. The cells were cultured at 37 °C until the OD_600_ reached 0.7, at which point protein expression was induced with 0.6 mM isopropyl-beta-thiogalactopyranoside (IPTG). The temperature was reduced to 16 °C and expression continued for 22 h. After harvesting (4 °C, 4000 rpm for 40 min), cell pellets were stored at –20 °C. For protein purification, the cells were resuspended in buffer A (50 mM Tris–HCl, 500 mM sodium chloride, 100 mM sodium sulfate, 5% v/v glycerol, 5 mM imidazole, 1 mM Tris-(2-carboxyethyl)-phosphine (TCEP), pH 7.5) with one tablet of EDTA-free protease inhibitor cocktail (Roche), 100 μg of lysozyme (Carl Roth), and 50 μg of deoxyribonuclease (DNAse) (New England Biolabs) per 1 L of original culture volume. Cell lysis was performed by sonication (50% power; 5 s pulse/10 s rest duty-cycle, for 20 min). The cell lysate was cleared by centrifugation (4 °C, 18000 rpm for 1 h). The supernatant was filtered and loaded onto a HisTrap HP column (GE Healthcare), washed first with buffer A and then with buffer A containing additional 2 M LiCl, before elution with a linear gradient of buffer A to buffer B (composition same as buffer A but with 300 mM imidazole). Nsp1-containing fractions of the eluate were exchanged back into buffer A using a desalting column, and then incubated with TEV protease (0.5 mg per 1 L of original culture volume) overnight at 4 °C. The cleaved Nsp1 was separated from the TEV protease and residual uncleaved protein by passing the dialysis mixture over the HisTrap column and washing with buffer A. The flow-through was collected, concentrated and loaded onto a HiLoad Superdex 75 16/600 size-exclusion column (GE Healthcare), previously equilibrated in buffer C (50 mM Tris–HCl, 500 mM sodium chloride, 100 mM sodium sulfate, 1 mM EDTA, 1 mM TCEP, pH 7.5). Pure Nsp1-containing fractions were identified by SDS-PAGE, pooled and exchanged into NMR buffer (50 mM sodium phosphate (pH 6.5), 200 mM sodium chloride, 2 mM dithiothreitol, 2 mM ethylene diamine tetra-acetic acid, 0.01% w/v sodium azide, 0.001% w/v 3-(trimethylsilyl)propane-1-sulfonate) by means of repeated dilution/concentration using Amicon centrifugal concentrators (10-kDa molecular-weight cutoff).

Uniformly ^15^N-labelled Nsp1-NTD (residues 13–127) was expressed in *E. coli* strain BL21 (DE3) in M9 minimal medium containing 1 g/L ^15^NH_4_Cl (Cambridge Isotope Laboratories) and 100 μg/mL ampicillin. Expression was induced at an OD_600_ of 0.7 with 1 mM IPTG for 18 h at 16 °C. Cell pellets were resuspended in Buffer D (50 mM Tris–HCl, 300 mM NaCl, 10 mM imidazole, 4 mM DTT and 100 µL protease inhibitor mix (SERVA), pH 8.0). The supernatant was cleared by centrifugation (40 min, 10000 g, 4 °C) and subsequently loaded onto a Ni^2+^-NTA gravity-flow column (Sigma Aldrich). Cleavage of the His_6_-GST-tag was achieved overnight at 4 °C with 0.5 mg of TEV protease per 1L of culture while dialyzing into Buffer D. The TEV protease and the cleaved tag were removed via a second Ni^2+^-NTA gravity-flow column. Further purification of Nsp1-NTD by size exclusion chromatography (HiLoad 16/600 SD 75, GE Healthcare) was carried out in Buffer E (25 mM NaPi, 250 mM NaCl, 2 mM TCEP, 0.02% w/v NaN_3_, pH 7.0). Fractions containing pure Nsp1-NTD were determined by SDS-PAGE, pooled and concentrated using Amicon centrifugal concentrators (3-kDa cutoff). Final NMR samples were prepared in Buffer E, containing 5% v/v D_2_O at Nsp1-NTD concentrations of 15 μM.

### NMR experiments

NMR samples (~ 550 uL total volume in 5-mm-diameter NMR tubes) were prepared with Nsp1 at a concentration of 300–400 uM, dissolved in NMR buffer. The protein appeared relatively stable over a period of several days, except for the gradual appearance of a set of small, sharp peaks characteristic of the dipeptides resulting from proteolysis. Appearance of these peaks was also accompanied by small shift-changes in the main set of peaks.

NMR experiments on full-length Nsp1 were recorded at 298 K on two Bruker Avance III-HD spectrometers running Topspin 3.2 software, with ^1^H field-strengths of 850 MHz and 600 MHz, and equipped with inverse HCN CP-TCI (helium-cooled) and CPP-TCI (nitrogen-cooled) cryogenic probeheads, respectively.

2D ^15^N-HSQC spectra were recorded using States-TPPI for frequency discrimination, with water suppression achieved via a combination of WATERGATE and water flip-back pulses to preserve the water magnetization (Bodenhausen & Ruben 1980; Piotto et al. 1992). Backbone resonance assignments were obtained from a standard-suite of 3D triple-resonance out-and-back-type spectra, comprising HNCO (Ikura et al. 1990; Kay et al. 1990), HN(CA)CO (Clubb et al. 1992; Kay et al. 1994), HNCA, HNCACB (Grzesiek & Bax 1992; Wittekind & Mueller 1993), HN(CO)CA and HN(CO)CACB (Bax & Ikura 1991; Yamazaki et al. 1994) spectra. With the exception of the HNCO experiment, which was recorded at both field-strengths, the through-carbonyl and the HN(CA)CO spectra were recorded at 600 MHz, while the remaining spectra were recorded at 850 MHz. Water suppression and frequency discrimination in the triple-resonance spectra were achieved as for the ^15^N-HSQC spectra.

For the Nsp1 NTD, 2D ^15^N-HSQC-TROSY experiments were measured at 950 MHz with acceleration of longitudinal ^1^H relaxation between scans via the Band-Selective Excitation Short-Transient (BEST) approach (Favier & Brutscher 2011; Lescop et al. 2007; Solyom et al. 2013)⁠ using exclusively shaped-pulses on ^1^H (bandwidth and offset of 4.5 and 8.5 ppm, respectively) and an inter-scan delay of 0.3 s.

NMR data were processed with NMRPipe v10.1 (Delaglio et al. 1995) and analysed in CcpNmr Analysis v2.4 (Vranken et al. 2005).

### Assignments and data deposition

**Fig. 1:**
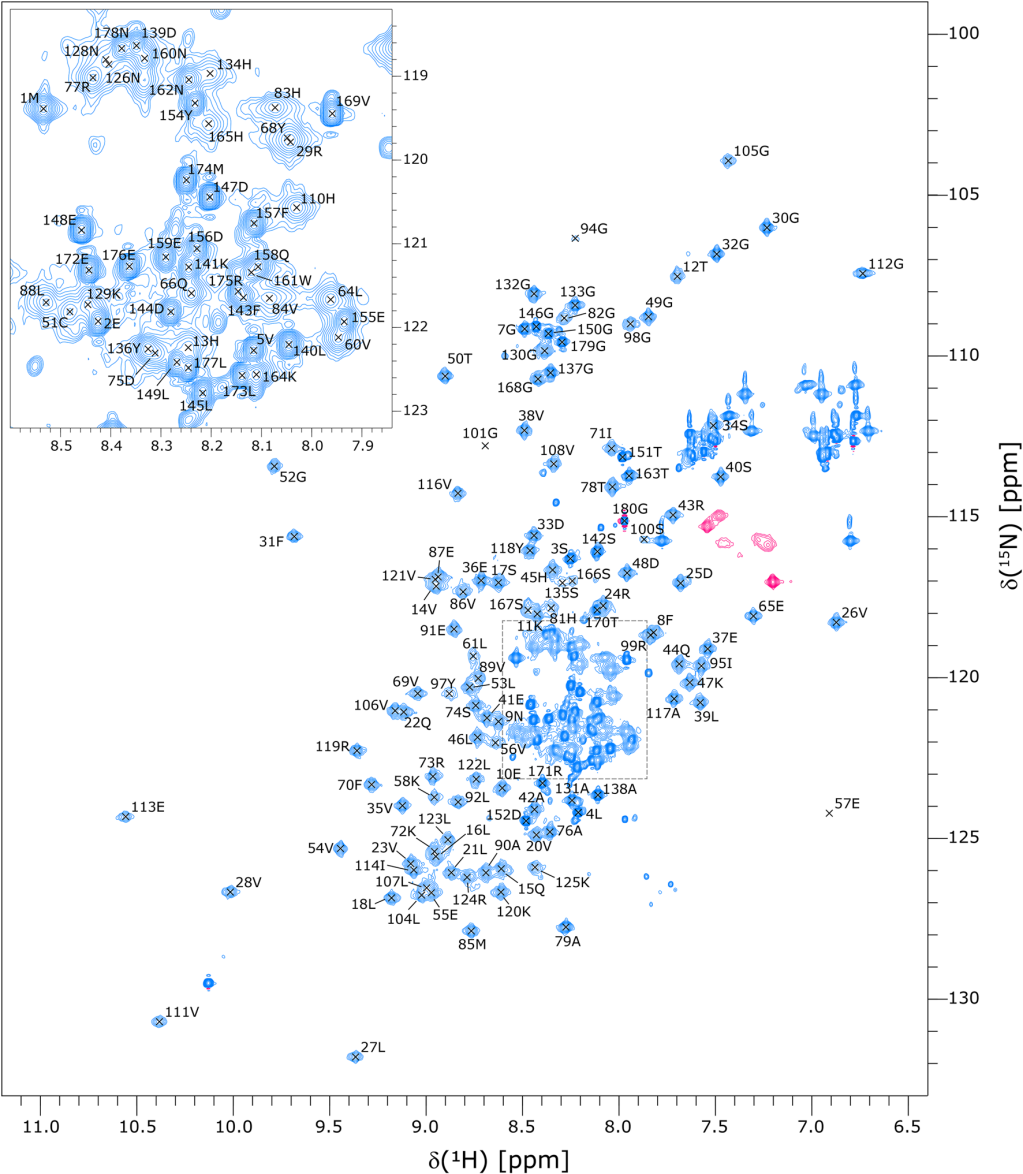
^15^N-HSQC spectrum of SARS-CoV-2 Nsp1 with backbone amide assignments. The excerpt in the top-left corner is an expansion of the crowded central region (corresponding to the dashed box in the main spectrum). The unlabeled peak at δ(^1^H) = 10.15 ppm, δ(^15^N) = 129.8 ppm corresponds to the side-chain N^ε1^–H^ε1^ group of residue 161W. The unlabeled pink peaks correspond to arginine side-chain N^ε^–H^ε^ groups, and are aliased from their true ^15^N chemical-shift positions at ~ 84–86 ppm. The peaks of the asparagine and glutamine side-chain amide groups are also unlabeled. The spectrum was recorded at a ^1^H field-strength of 850 MHz

The ^15^N-HSQC spectrum of SARS-CoV-2 full-length Nsp1 has the appearance expected for a protein comprising a well-folded globular domain together with an extended and largely disordered tail (Fig. 1). The amide peaks from the residues in the globular domain are widely dispersed with a ^1^H chemical-shift dispersion of ~ 4 ppm, while the peaks from the C-terminal region are clustered more closely together with ^1^H chemical-shifts in the range 7.9–8.5 ppm. In general, the peaks from the C-terminal region are much sharper and more intense than the more widely dispersed peaks from the globular domain, as would be expected for a disordered tail. Differential linewidths and intensities characteristic of exchange-mediated line-broadening effects were observed within both the C-terminal region and the globular domain. Some residues in the C-terminal region and towards the C-terminal end of the globular domain appeared to give rise to multiple peaks, with one or two weak-intensity peaks in addition to a strong-intensity peak. The weak-intensity peaks are probably due to low-populated alternate conformers associated with cis-geometry amide bonds at proline residues.

We assigned the backbone resonances to a high-degree of completeness, obtaining ^1^H and ^15^N assignments for 165 of the 172 assignable backbone amide groups (96.0%), and ^13^C assignments for 96.1%, 97.2% and 97.5% of the C', C^α^ and C^β^ nuclei, respectively. Amide assignments are missing for residues 59G, 63Q, 93E, 96Q, 102E, 103 T and 127G. These residues are located in four stretches for which the assigned amide peaks are significantly broader and weaker. The amide signals for the missing residues are probably so broadened that the corresponding peaks simply do not appear above the noise in the ^15^N-HSQC spectrum, and certainly would not yield detectable peaks in the 3D triple-resonance spectra.

**Fig. 2:**
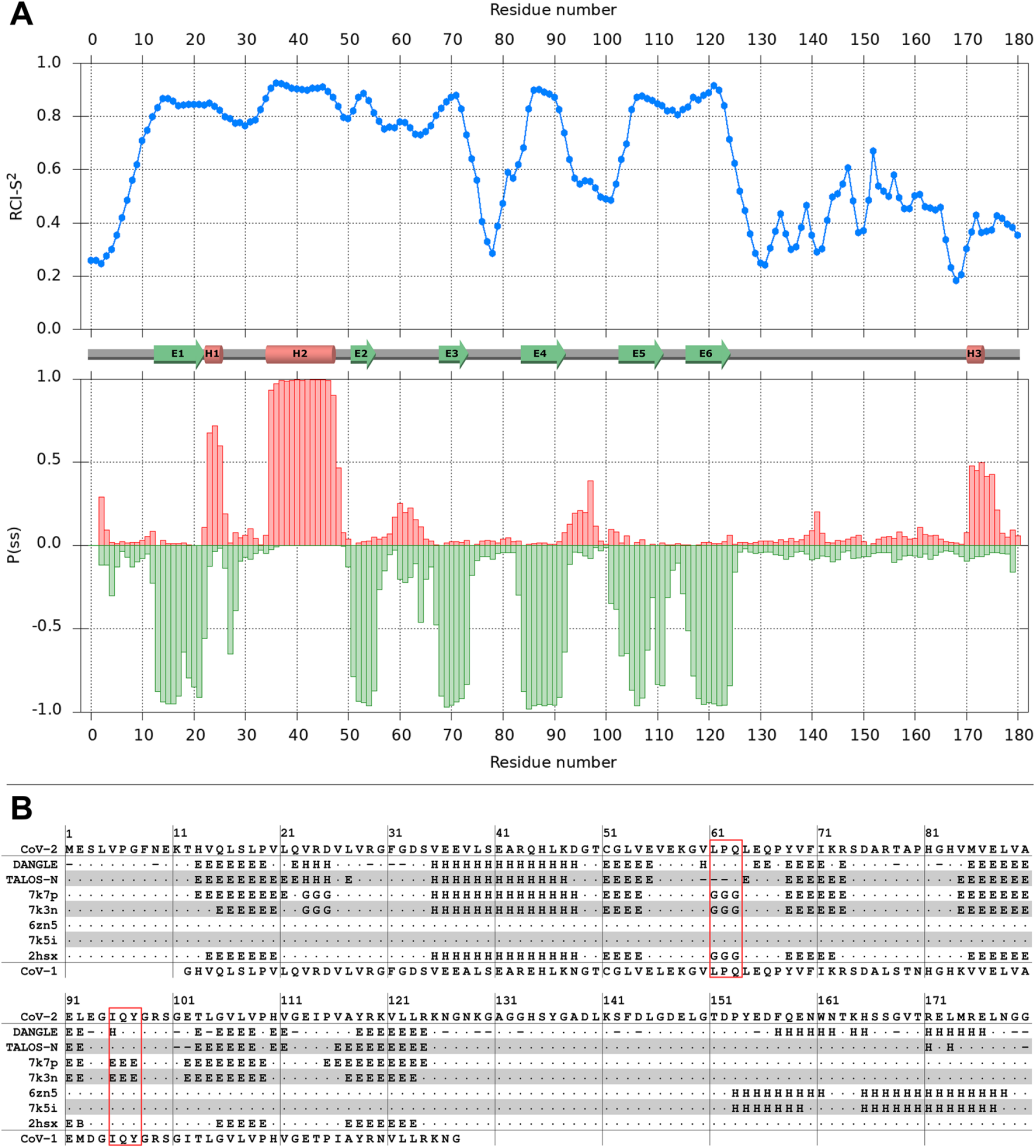
Secondary-structure of SARS-CoV-2 Nsp1. **a**. Results from TALOS-N analysis of the assigned backbone chemical shifts. Top: random-coil-index-derived squared-order-parameters (RCI-S^2^) plotted by residue number. Bottom: probabilities of helical (positive, red) and strand (negative, green) secondary-structure plotted by residue number. The schematic secondary-structure shown between the two plots is based on the TALOS-N prediction. **b**. Comparison of the DANGLE- and TALOS-N-predicted secondary-structures for SARS-CoV-2 Nsp1 with those of the crystal structures of the globular domain (PDB codes 7k7p & 7k3n), the cryo-EM structures of the C-terminus in complex with the small ribosomal subunit (PDB codes 6zn5 & 7k5i) and the NMR structure of the globular domain of SARS-CoV-1 Nsp1 (PDB code 2hsx). The two regions where secondary-structure elements present in the crystal structures are missing from the chemical-shift-based predictions are highlighted in red boxes. The secondary structures of the PDBs were calculated with STRIDE (Frishman & Argos 1995). The Nsp1 sequences from SARS-CoV-2 and SARS-CoV-1 (shown above and below the secondary-structure annotations, respectively) were aligned with ClustalW (Sievers et al. 2011). The codes for the secondary-structure annotations are as follows: ‘E’ denotes extended β-strand; ‘H’ denotes either generic helix (DANGLE & TALOS-N) or specific α-helix (STRIDE); ‘G’ denotes 3_10_ helix (STRIDE only)

The assigned backbone chemical shifts were used to predict the secondary-structure with DANGLE (Cheung et al. 2010) and TALOS-N (Shen & Bax 2013), and also to calculate random-coil-index-derived order-parameters (RCI-S^2^) (Berjanskii & Wishart 2005). Panel A of Fig. 2 shows the RCI-S^2^ values and TALOS-N-derived helix/strand probabilities. Panel B shows the TALOS-N- and DANGLE-predicted secondary structures of SARS-CoV-2 Nsp1 together with the secondary-structure assignments of the two crystal structures of the folded globular domain (PDB codes 7k7p and 7k3n), the two cryo-EM structures of the C-terminus bound to the small ribosomal subunit (PDB codes 6zn5 (Thoms et al. 2020) and 7k5i), and the solution-NMR structure of Nsp1 from SARS-CoV-1 (PDB code 2hsx).

The well-folded globular domain of Nsp1 extends approximately from residue 10 to residue 125, and consists of six β-strands and two helices (labelled E1–E6 and H1–H2 in Fig. 2, respectively). The two long loops between strands E3 and E4, and between E4 and E5 show relatively low order-parameters, indicative of significant internal flexibility. Overall, the chemical-shift-derived secondary-structure predictions for the globular domain are very similar to the secondary structures of the two crystal structures and the solution-structure of Nsp1 from SARS-CoV-1. The short proline-containing 3_10_ helix (residues 61–63) and the short β-sheet formed by residues 95–97 observed in the crystal structures are not predicted from the backbone chemical shifts, but some assignments were missing for residues in these two regions, compromising the ability to make accurate chemical-shift-based secondary-structure predictions.

In full-length Nsp1, the C-terminal region from residue 126 onwards is partially but not completely disordered, with RCI-S^2^ values falling mostly in the range 0.3–0.6, and very little canonical secondary-structure propensity for residues 125–155. DANGLE predicts two additional helices at the very C-terminus (residues 157–162 and 171–176). While the TALOS-N helical assignments in the same sequence-stretch are limited to just two residues (171 & 173; labelled H3 in Fig. 2), the corresponding probabilities indicate a clear helical propensity for residues 171–175. Interestingly, the region of helical propensity at the very end of the C-terminus corresponds approximately to the two helices observed in the cryo-EM structure of Nsp1 interacting with the small ribosomal subunit, suggesting that these helices, which are partially formed in free Nsp1, become stabilized upon insertion into the mRNA tunnel of the small ribosomal subunit.

**Fig. 3:**
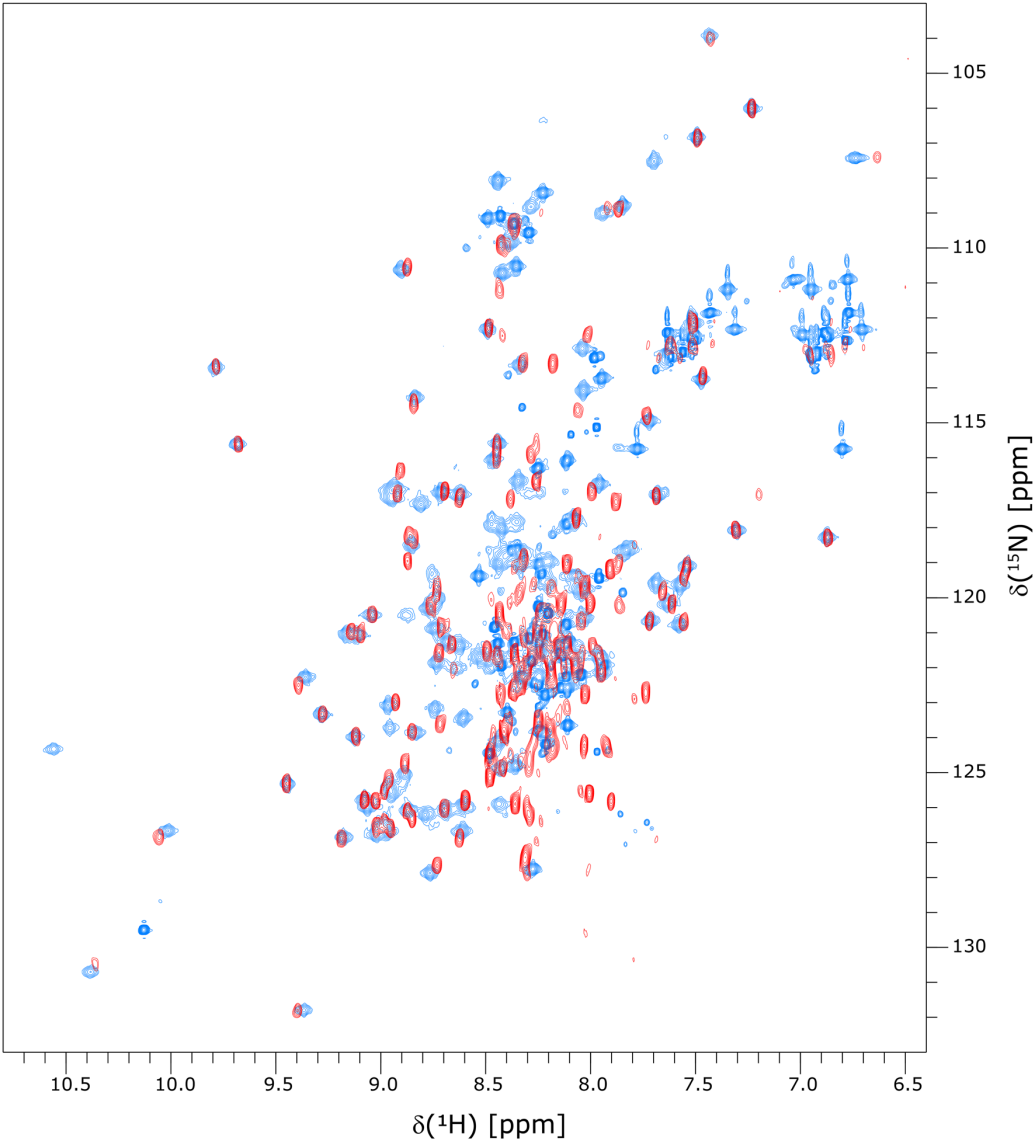
Overlay of ^1^H,^15^N correlation spectra of full-length Nsp1 and the Nsp1 NTD (residues 13–127). Overlay of the ^15^N-HSQC-TROSY spectrum of Nsp1 NTD (red; recorded at 950 MHz) with the ^15^N-HSQC spectrum of full-length Nsp1 (blue; recorded at 850 MHz). The signal-to-noise ratios of the two spectra are not quantitatively comparable due to differences in concentrations and acquisition parameters

Based on the strong sequence-similarity with SARS-CoV-1 Nsp1, we also investigated the isolated Nsp1 NTD (residues 13–127). Unexpectedly, the protein showed little solubility and we were not able to achieve high concentrations in NMR-compatible buffers, although the domain boundaries are in agreement with the available crystal and NMR structures of SARS-CoV-1 Nsp1 (Fig. 2b). Nevertheless, we were able to record 2D ^1^H,^15^N correlation spectra. An overlay of the ^15^N-HSQC-TROSY spectrum of Nsp1 NTD with the ^15^N-HSQC spectrum of full-length Nsp1 (Fig. 3) shows reasonable agreement for the residues of the NTD, although there are some chemical shift differences and also instances of relative line-broadening for the Nsp1 NTD peaks. It is conceivable that in the full-length protein, the disordered C-terminal domain interacts with the NTD, thereby stabilizing its fold and improving solubility. We are currently conducting further experiments to investigate this hypothesis.

## Data Availability

The ^1^H, ^13^C and ^15^ N backbone chemical-shift assignments of SARS-CoV-2 Nsp1 have been deposited at the BioMagResBank (https://www.bmrb.wisc.edu) under accession number 50620.
